# Blasto in the Bronx: An Unusual Case of Severe Cutaneous Blastomycosis in New York City

**DOI:** 10.7759/cureus.80251

**Published:** 2025-03-08

**Authors:** Sabirah N Kasule, Andrea K Thet, Srikaran Bojja, Abolfazl Sodagar, Joshua Davidson, Donald D Rudikoff, Lendelle Raymond, Cosmina B Zeana

**Affiliations:** 1 Infectious Disease, BronxCare Health System, New York, USA; 2 Infectious Disease, HCA Florida JFK Hospital, Atlantis, USA; 3 Geriatrics, Rutgers New Jersey Medical School, Rutgers University, Newark, USA; 4 Internal Medicine, BronxCare Health System, New York, USA; 5 Pulmonary and Critical Care, BronxCare Health System, New York, USA; 6 Dermatology, BronxCare Health System, New York, USA; 7 Pharmacy, BronxCare Health System, New York, USA

**Keywords:** blastomycosis, cutaneous, fungal, infection, new york city, nyc

## Abstract

Urban cases of blastomycosis are extremely rare and usually involve an individual or individuals with recent travel to, and outdoor exposure in, an endemic area. We report a case of severe cutaneous blastomycosis in a New York City resident. He presented to our hospital in 2023 with multiple cavitary lung nodules and a large area of fungating lesions on his back that had been progressing for a month. Cultures and pathology from the skin lesions confirmed a diagnosis of cutaneous blastomycosis, and the patient was successfully treated with a prolonged course of oral itraconazole. Given no travel history outside of the five boroughs, we believe the patient acquired the infection endemically and is the first published case of blastomycosis acquired in New York City.

## Introduction

*Blastomyces sp.* are dimorphic fungi native to the Ohio, Mississippi, and St. Lawrence River valleys [1]. An endemic focus in New York state was not seriously considered until 2018 when the New York State Department of Health (NYSDOH) and the Centers for Disease Prevention and Control (CDC)’s statewide review found a high incidence of blastomycosis in a county of the Capital District in Upstate New York (CDNY) [1].

To date, however, this is the first reported case of blastomycosis acquired in New York City (NYC). In a previously published case from 2023, the individual had a significant history of travel to Colorado a month prior to presentation in NYC [2]. Our patient’s diagnosis was made through a combination of pathology findings from skin biopsy, fungal cultures of the skin lesions, blastomyces serology, and blastomyces quantitative urine antigen. One month after the initiation of oral itraconazole, the patient had significant improvement in his skin infection. This case suggests that the endemic focus of blastomycosis in New York State likely extends beyond CDNY, and more work is needed to define its borders. Practitioners should consider blastomycosis in the differential diagnosis of atypical lung and skin lesions, as prompt diagnosis could improve outcomes.

## Case presentation

A 49-year-old male presented to our hospital in July 2023 with a one-month history of a thick rash on his back. The rash began as a pruritic in-grown hair that later became infected from incessant scratching. Over the next few weeks, the infection spread to the rest of his back as multiple skin lesions, one of which began to discharge pus two days prior to admission. His past medical history was notable for hidradenitis suppurativa of the axilla, a 10-pack-a-year smoking history, and pulmonary nodules diagnosed during an emergency department (ED) visit for community-acquired pneumonia (CAP) in 2022. The review of systems was otherwise negative.

**Figure 1:**
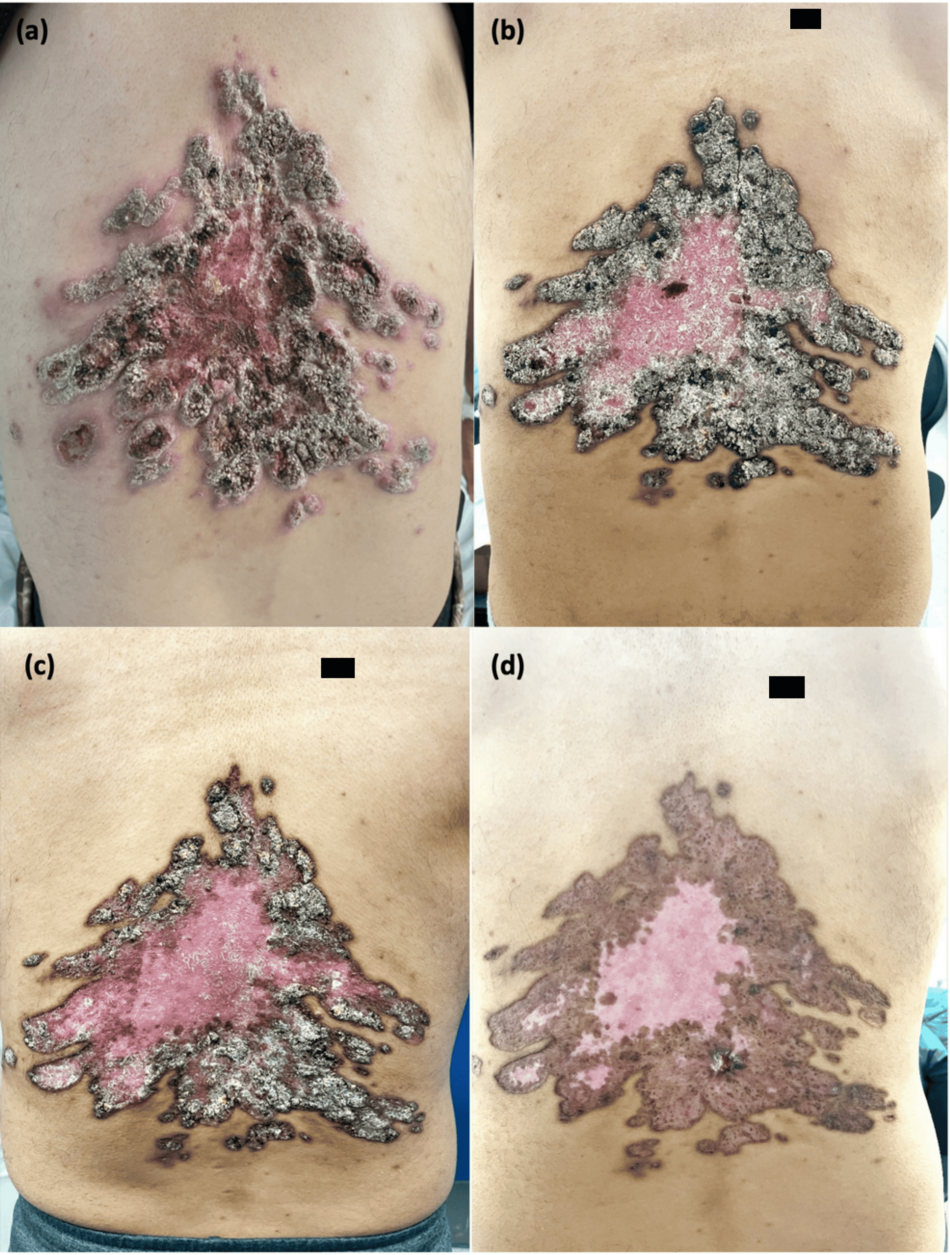
(a) Lesions at presentation: a 20×20 cm hyperkeratotic and fungating mass with an open abscess cavity on the left side; (b) lesions one month after starting itraconazole; (c) lesions two months after initiating itraconazole; (d) lesions five months after initiating itraconazole.

On presentation, he was afebrile and hemodynamically stable. He had a normal white blood cell count of 10.7 k/uL (normal range: 4.8-10.8 k/uL), a sedimentation rate of 75 (normal range: <30), a C-reactive protein of 108.78 (normal range: <3), a glucose of 444 mg/dL (normal range: 70-120 mg/dL), and a glycated hemoglobin (A1c) of 12.6% (normal range: 4.7-6.4%). Examination of the mid-back revealed multiple excoriated lesions that coalesced into a hyperkeratotic, fungating, and verrucous rash with raised erythematous borders. On the left side of the 20 x 20 cm area, yellow-colored pus actively drained from an open abscess cavity (Figure 1a). He was empirically placed on intravenous vancomycin and piperacillin-tazobactam for suspected bacterial skin and soft tissue infection. Insulin was also initiated for newly diagnosed type II diabetes.

The initial culture of the drainage grew *methicillin-sensitive Staphylococcus aureus* (MSSA). A preliminary diagnosis of blastomyces-like pyoderma was made, and a shave biopsy of the lesions was performed on hospital day 2 for confirmation. Additional history collected at this time revealed that the patient was born and raised in the Bronx but worked as a door attendant in Manhattan. He had never left NYC and stated that “if I can’t get there on the 4 train, I’m not going”. He denied any outdoor hobbies. He had no pets or other animal exposures. He did not regularly engage with the healthcare system and had never been incarcerated. He had not recently purchased any furniture or clothing. Screenings for HIV and syphilis, a QuantiFERON-TB gold plus test, three sputum cultures for mycobacteria, and multiple bacterial and fungal blood cultures were ultimately negative.

**Figure 2:**
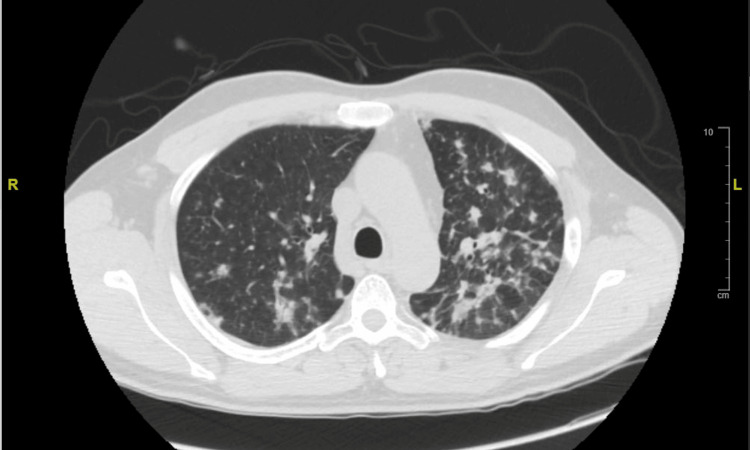
CT chest from the 2023 admission showing multiple ill-defined nodular infiltrates scattered throughout both lungs, predominantly in the upper lobes. Several nodules exhibit cavitation.

**Figure 3:**
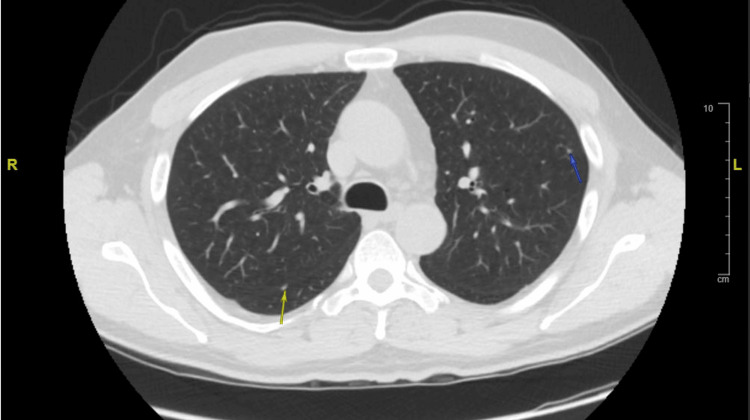
CT chest from 2022 showing a 3 mm left upper lobe nodule (blue arrow) and a 5 mm perifissural right lower lobe nodule (yellow arrow). Not pictured are a 6 mm perifissural right lower lobe nodule and a large left lower lobe consolidation, which were also present.

Due to his history of pulmonary nodules, a computed tomography (CT) of the chest was performed, revealing multiple, ill-defined, nodular infiltrates scattered throughout both lungs. These nodules predominated in the upper lobes, and several exhibited cavitation (Figure 2). Findings had developed since his previous CT chest in 2022, which only showed a small nodule in the left upper lobe, two nodules in the right lung consistent with benign peri-fissural nodules, and a left lower lobe infiltrate (Figure 3). Infectious diseases (ID) recommended broad evaluation for fungal and mycobacterial pathogens, given the unusual skin and lung findings. Pulmonology additionally planned for bronchoscopy with bronchoalveolar lavage (BAL) to rule out Langerhans cell histiocytosis and malignancy, but, unfortunately, the patient self-directed his own discharge. He was given an additional 21-day course of amoxicillin-clavulanate for the skin infection.

**Figure 4:**
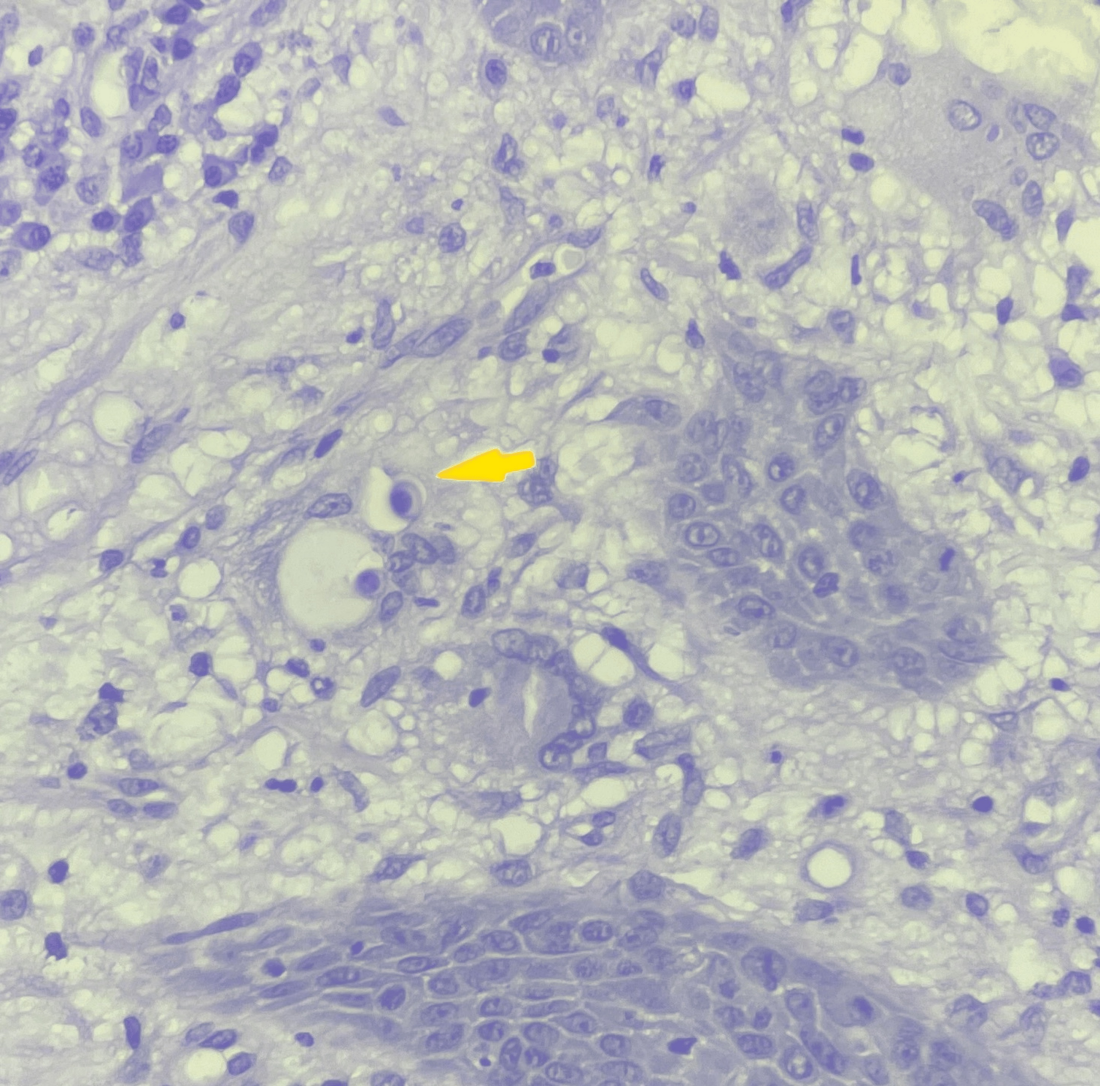
Skin biopsy with Periodic acid-Schiff (PAS) stain showing thick-walled yeasts with broad-based budding.

He eventually underwent an outpatient fiberoptic bronchoscopy and BAL. Bacterial cultures from the BAL grew *Streptococcus parasanguinis*. Fungal and mycobacterial cultures were negative, as was the BAL polymerase chain reaction (PCR) for *Blastomyces* and *Histoplasma* species.​​​​ Lung biopsy showed no yeast forms resembling *Blastomyces sp*. Meanwhile, the patient’s blastomyces serology and blastomyces urinary antigen returned positive. His histoplasma urinary and serum antigens were both weakly positive, but his histoplasma serologies were negative. Pathology from the shave biopsy showed skin with acute, chronic, and granulomatous inflammation with micro-abscess formation. A periodic acid-Schiff (PAS) stain showed thick-walled yeasts with broad-based budding, consistent with *Blastomyces sp. *(Figure 4). Fungal culture of the biopsy grew a mold after 15 days of incubation, which was identified as *Blastomyces dermatitidis/gilchristi* by DNA-sequencing.

Table 1 summarizes all diagnostic testing that was done to confirm the diagnosis of blastomycosis. All tests were performed at the Quest Diagnostics laboratory, NYC, except for the blastomyces urinary antigen, which was performed at ARUP labs, Salt Lake City, Utah, and the *Histoplasma/Blastomyces sp.* PCRs in the BAL were performed at the Mayo Clinic Laboratories in Rochester, MN.

**Table 1:** Diagnostic testing performed to confirm the diagnosis of blastomycosis. MSSA: *methicillin-sensitive Staphylococcus aureus*; PAS: Periodic acid-Schiff; N/A: not applicable; EIA: enzyme immunoassay; BAL: bronchoalveolar lavage; PCR: polymerase chain reaction

Source	Culture/microbiology/pathology	Value (interpretation)	Normal range
Sputum	Mycobacteria culture with fluorochrome (x3)	No growth	No growth
Skin biopsy	Aerobic/Anaerobic culture (x3)	Heavy growth of MSSA	No growth
Skin biopsy	Fungal culture	Mold	No growth
Skin biopsy	DNA sequencing of mold	Blastomyces dermatitidis/gilchristi	Negative
Skin biopsy	Mycobacterial culture	No growth	No growth
Skin biopsy	Pathology	Acute, chronic, and granulomatous inflammation with micro-abscess formation	N/A
Skin biopsy	Pathology: PAS special stain	Thick-walled yeasts, some are broad-based budding	N/A
Blood	Blastomyces serology	Detected	Not detected
Blood	Histoplasma serology	Not detected	Not detected
Blood	Histoplasma serum ag	0.32 ng/mL (positive)	<0.2 ng/mL
Blood	1,3 beta-D glucan	<31 pg/mL (negative)	<60 pg/mL
Blood	Aerobic/Anaerobic culture (x2 sets)	No growth	No growth
Blood	Mycobacterial culture (x1)	No growth	No growth
Blood	Fungal culture (x3)	No growth	No growth
Serum	Aspergillus antigen, EIA	0.06 (negative)	<0.50
Urine	Blastomyces urinary antigen	21.6 U/mL (positive)	<1.25 U/mL
Urine	Histoplasma urinary antigen	0.4 ng/mL (positive)	<0.2 ng/mL
BAL	Aerobic/Anaerobic culture	Scant growth of *Streptococcus parasanguinis*	No growth
BAL	Fungal culture	No growth	No growth
BAL	Mycobacterial culture	No growth	No growth
BAL	Histoplasma/BlastomycesPCR	Negative	Negative

The case was discussed with the NYSDOH and CDC, and immediately post-BAL, the patient was initiated on a loading dose of 200 mg of an oral itraconazole solution three times daily for three days, followed by a maintenance dose of 200 mg twice daily. The solution was picked over the capsule because of its superior bioavailability. Intravenous liposomal amphotericin B was considered, but the patient deferred further admissions. Additionally, his potential pulmonary involvement was minimal to moderate. The *S. parasanguinis* recovered from the BAL was presumed to be related to aspiration in the setting of poor dental hygiene. Given that it was covered by the amoxicillin-clavulanate that he had received for his superimposed MSSA skin infection, no further changes to management were made.

The patient’s skin significantly improved on follow-up visits with ID at one, two, and five months after initiation of itraconazole (Figures 1b-1d). He continued to have no pulmonary symptoms. A repeat CT chest six months after treatment showed almost complete resolution of his cavitary pulmonary nodules. He successfully completed seven months of treatment before being lost to follow-up.

## Discussion

The traditional epidemiological boundaries of *Blastomyces sp.* include the Midwestern and Southeastern states along the Mississippi and Ohio River valleys and the Northeastern states and Canadian provinces along the St. Lawrence River valley [1]. More recently, upstate New York was added to the roster after a CDC and NYSDOH statewide review of cases in the region [1]. Prior to this, cases were only intermittently reported, including two cases of fatal systemic blastomycosis in Rochester, NY, in the 1930s [3] and three cases of pulmonary blastomycosis in Central New York State between 2012 and 2013 [4].

Following the NYSDOH and CDC’s 2018 review, Bethuel et al. published a series of eight cases of pulmonary blastomycosis diagnosed at a rural hospital in CDNY between 2017 and 2019. Only one case had travel to an endemic region. All resided in a rural area along the Susquehanna River [5]. Later, Austin et al. (2021) reviewed 20 cases of blastomycosis seen at the Albany Medical Center and Albany Stratton Veterans Affairs Center between 2000 and 2019. Ninety percent of their patients lived in CDNY and 65% within the Mohawk River valley [6]. More recent reports include two cases of pulmonary blastomycosis in the Adirondack’s region in 2019 [7], a case in 2020 of osseous and cutaneous blastomycosis in upstate NY [8], and a case of pulmonary blastomycosis in 2022 that was complicated by severe acute respiratory distress in an orthotopic liver transplant patient from Amsterdam, NY [9]. Relevantly, these cases were primarily in rural locations or had typical occupational or recreational exposures. Reasons for this surge in cases have included flooding of the major waterways [5], but recent data also suggests that the original epidemiological boundaries of endemic fungi have been shifting worldwide due to multiple factors, including climate change [10].

The typical patient with blastomycosis is a male between the ages of 25 and 50 who engages in outdoor activities. Nearly all cases begin as a pulmonary infection through inhalation of aerosolized conidia, the infectious form. Approximately 50% of infected patients will remain asymptomatic. The rest typically present with pneumonia, characterized by an alveolar or mass-like infiltrate on radiography [11]. Cutaneous lesions usually occur secondarily through dissemination, though there are cases of primary cutaneous infection through direct inoculation. Lesions appear more frequently on the face, neck, and extremities and manifest as verrucous plaques or cutaneous ulcers, often accompanied by a distinctive purple-blue halo. These lesions may develop suppuration and spontaneous drainage, resulting in deep cutaneous ulcers. Micro-abscesses are a common finding on biopsy [12].

Our patient’s case is unique in that he did not have typical travel, occupational, or recreational exposure to the fungus’ preferred environment. He later divulged that his mother loved potted plants and kept many of them in his house. We hypothesize that the soil from these plants could have been the nidus for his infection. It is possible that he inhaled aerosolized conidia and had a mild pulmonary course, but, potentially because of unrecognized diabetes, the fungus disseminated to the skin. BAL cultures could have been negative because of sampling error. Alternatively, it is possible that he had primary cutaneous blastomycosis inoculated after persistent scratching, and the lung nodules’ presence and resolution were unrelated. Once his cutaneous infection was established, it likely became super-infected with MSSA, which explains its heavy growth from the draining lesions.

The primary diagnostic methods for blastomycosis involve the visualization of the organism in tissue specimens stained with methenamine silver or PAS stains. Culture, which is positive in almost all cases, should be performed and typically shows mold growth within two to four weeks on various culture media, including Sabouraud agar, at room temperature [13]. Blastomyces serology and blastomyces urinary antigen can provide additional confirmation. Histoplasma antigens can cross-react in the setting of blastomycosis [14]. This is the most likely explanation for the weakly positive histoplasma urinary and serum antigens in our case. It is important to note that the genus *Blastomyces* primarily consists of two fungi, *B. dermatitidis* and *B. gilchristi*. They are indistinguishable by phenotype and by commercially available molecular probes. Identification at the species level often requires PCR followed by specialized sequence analysis [15]. Our patient had broad-based budding yeast on biopsy, grew a mold in 15 days on culture, and had a positive PCR for *B. dermatitidis/gilchristi*. At the time of publication, however, no further delineation was done.

Liposomal amphotericin B and itraconazole are the drugs of choice for blastomycosis. Itraconazole is the preferred agent for mild to moderate disease without central nervous involvement due to its milder side-effect profile [11]. Severe disease, including symptomatic pulmonary infection with dissemination, requires intravenous liposomal amphotericin B [11]. Our patient’s disease was considered mild to moderate and readily responded to oral itraconazole.

## Conclusions

Blastomycosis should be considered in patients residing in NYC who present with unusual pulmonary or cutaneous symptoms. Classic exposures to the outdoors and/or decaying matter may not be present, but this should not rule out the disease, as early recognition could improve outcomes. Monotherapy with oral itraconazole remains a suitable management for mild to moderate disease.
